# Sex-specific differences in zebrafish brains

**DOI:** 10.1186/s13293-022-00442-2

**Published:** 2022-06-17

**Authors:** Gang Zhai, Jingyi Jia, Ceyhun Bereketoglu, Zhan Yin, Ajay Pradhan

**Affiliations:** 1 State Key Laboratory of Freshwater Ecology and Biotechnology, Institute of Hydrobiology, Chinese Academy of Sciences, Wuhan, 430072 Hubei China; 2 Department of Bioengineering, Faculty of Engineering, Marmara University, 34854 Istanbul, Turkey; 3 Biology, The Life Science Center, School of Science and Technology, Örebro University, 70182 Örebro, Sweden

**Keywords:** Steroid hormones, Gonads, Differentiation, Disease, Neurons

## Abstract

In this systematic review, we highlight the differences between the male and female zebrafish brains to understand their differentiation and their use in studying sex-specific neurological diseases. Male and female brains display subtle differences at the cellular level which may be important in driving sex-specific signaling. Sex differences in the brain have been observed in humans as well as in non-human species. However, the molecular mechanisms of brain sex differentiation remain unclear. The classical model of brain sex differentiation suggests that the steroid hormones derived from the gonads are the primary determinants in establishing male and female neural networks. Recent studies indicate that the developing brain shows sex-specific differences in gene expression prior to gonadal hormone action. Hence, genetic differences may also be responsible for differentiating the brain into male and female types. Understanding the signaling mechanisms involved in brain sex differentiation could help further elucidate the sex-specific incidences of certain neurological diseases. The zebrafish model could be appropriate for enhancing our understanding of brain sex differentiation and the signaling involved in neurological diseases. Zebrafish brains show sex-specific differences at the hormonal level, and recent advances in RNA sequencing have highlighted critical sex-specific differences at the transcript level. The differences are also evident at the cellular and metabolite levels, which could be important in organizing sex-specific neuronal signaling. Furthermore, in addition to having one ortholog for 70% of the human gene, zebrafish also shares brain structural similarities with other higher eukaryotes, including mammals. Hence, deciphering brain sex differentiation in zebrafish will help further enhance the diagnostic and pharmacological intervention of neurological diseases.

**Supplementary Information:**

The online version contains supplementary material available at 10.1186/s13293-022-00442-2.

## Introduction

In mammals, the development of the gonads begins with an undifferentiated gonad (bipotential gonad) that can develop into either a testis or ovary [1]. The decision to follow the testis or ovary pathway is primarily governed by the chromosome constitution (XX/XY), which was established while studying human sex-related diseases [2, 3]. In males, the presence of the *sex determining region Y* (*SRY*) gene on the Y chromosome drives testis differentiation [1, 4–6]. In the absence of *Sry*, ovarian development continues [1, 4] and consequently, ovarian differentiation is suggested to be the default pathway [7]. However, as indicated by Yao [8], it is premature to suggest that the ovary is a default pathway, as it could have its own active genetic or signaling pathway to regulate development. In addition, the bipotential gonad has also been observed in several fish species before sex differentiation, and several sex determination genes have also been identified, including *dmY* [9], *gsdfY* [10], *sox3Y *[11], *amhy* [12, 13], *amhr2* [14], *gdf6Y* [15], *dmrt1* [16, 17], and *sdY* [18, 19]. Other sex-differentiation mechanisms further improve our understanding of the gonadal sex differentiation process. It has been argued that the downstream signaling cascades and genes in zebrafish and other fish species show a pattern similar to that observed in mammals (reviewed in [20–23]). Many studies using exogenous compounds (hormones and chemicals) [24–28], morpholinos [29–32], and gene editing [33–38] have identified important genes, pathways and critical periods of gonadal development in fish species. Hence, the absence of a strong master regulator gene such as *Sry* in fish species allows for the easy manipulation of the sex differentiation outcome and in-depth study of the process.

The developing gonads secrete the steroid hormones testosterone (T) and estradiol (E2), which further differentiate the sex organs. The secreted T and E2 also organize the brain neuronal networks in a male- or female-specific manner. Similar to the ovary, the female brain has been suggested to be the default developmental pathway [39]. On the other hand, in males, testosterone masculinizes the developing brain, either directly through the activation of the androgen receptor (AR) or indirectly via the stimulation of estrogen receptors (ER) following the conversion of T into E2 [3, 40].

**Fig. 1:**
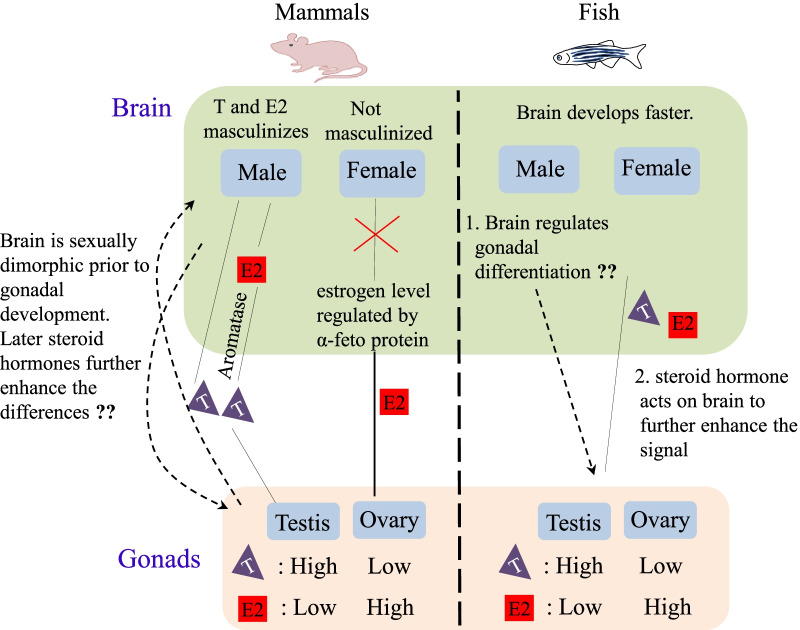
Brain sex differentiation. The steroid hormones synthesized in the gonads act on the brains to differentiate it into male and female types. In mammals, it is also indicated that the gene expression in developing brains is sexually dimorphic prior to the action of gonadal hormones. This suggests that the early brain could signal the gonads, or it could pre-condition the brain so that testosterone (T) and estradiol (E2) act differentially in male and female brains. The brain sex differentiation process in fish species, including zebrafish is largely unknown. Unlike mammals, fish are born with a developed brain, which helps them to forage and escape predators. This could indicate that the developing fish brain signals the gonad to develop into either a testis or ovary and later the steroid hormones synthesized by the gonads further enhances the sex-specific differences in the brain

Recent findings suggest that human male and female brains display differential connectomic, methylomic and transcriptomic profiles [41, 42]. Despite extensive advances in neuroscience, the molecular mechanisms underlying these differences remain unclear. Pfeiffer in the 1930s and Phoenix et al. in the 1950s provided important information on brain sex differentiation. Pfeiffer transplanted ovaries into adult mice and noted cyclical activity only in mice that were castrated in the neonatal stage and not in the adult stage [43]. Phoenix et al. [3] working with guinea pigs, demonstrated that treating prenatal female guinea pigs with testosterone resulted in typical male behavior in adults, but this effect was not observed when testosterone was administered during the perinatal or adult stages. These and other studies have resulted in the formulation of the classical model of brain sex differentiation, which suggests that the gonadal steroid hormones (androgens and estrogens) are the main drivers for establishing male and female neural networks [3, 40]. The classical concept of brain sexual differentiation states that the chromosomal constitution (XX or XY) determines the type of gonad, and the hormones secreted by these organs program the brain neural network differently [3, 40]. In males, T directly or indirectly following conversion to E2 by aromatase results in the masculinization of the brain. In females comparatively high levels of alpha-fetoprotein bind to estrogen and protect the brain from masculinizing events [44–46] (Fig. 1).

As indicated by Arnold and Breedlove [47], the organization and activation effect of steroid hormones (the classical theory) needs to be reevaluated as many elements are not taken into account. The classical model does not provide a complete picture, as the genetic differences are not considered; more importantly, hormones may not be the early determinant of sexual differentiation. There is evidence that prenatal brains can show sex-specific differences [48, 49]. Studies on mice and chickens indicate that the developing brains display differential gene expression prior to gonadal hormone action in the brain [48, 49]. This indicates that there are genetic differences in developing brains and that these differences result in the differential development of male and female brains. *Sry* is a master regulator gene that determines testis fate [4]. *Sry* expression can be detected at approximately 10.5 days post coitum (dpc) and peaks at 11.5 dpc in mouse gonads to initiate testis differentiation [50]. In the brain, *Sry* expression co-localizes with tyrosine hydroxylase, an enzyme involved in dopamine synthesis in the *substantia nigra.* The downregulation of *Sry* in the brain leads to reduced expression of tyrosine hydroxylase, suggesting a positive regulation by Sry [51]. The sex chromosomal constitution (XX or XY) of brain cells could also contribute to sexual dimorphism, as it has been reported that XXY and XYY boys develop testes, but show different social behaviors than XY boys [52]. A study on zebra finches showed that a hormone treatment in females could not fully masculinize the song center [53]. An aromatase inhibitor treatment induced testicular tissue in the genetic female zebra finches; however, the song system remained completely feminine [54]. The gynandromorphic zebra finch has one-half of its brain and body genetically male, and the other half is genetically female. Despite a bilateral body where the brain is under the influence of the same gonadal hormone, the finch still develops a histologically identifiable song center on the male side of the brain, whereas the female side of the brain remains feminine [55]. These studies further support the notion that genetic differences also play an essential role in orchestrating sex-specific neuronal networks apart from gonadal steroid hormones.

In contrast to mammals, where the gonads develop first and the secreted steroid hormones organize the brain, the opposite could be true in teleosts. This indicates that brain development prior to gonadal development is important for teleosts as they are independent right from the start and this could mean that the brain will determine the fate of the gonadal development [56]. However, recent studies on mouse and chicken brains indicate that the developing brains have sex-specific differences prior to the action of gonadal steroids on the brain [48, 49]. In zebrafish, depending on the expression of brain-specific aromatase (*cyp19a1b*), larvae can be segregated into high- and low-expression groups prior to gonadal differentiation [57–59]. However, these studies did not determine the sex of these two populations, and used the whole embryo for gene analysis, which could confound the analysis owing to signals from other tissues. Nonetheless, these studies indicate that the zebrafish brain exhibits sex-specific differences before gonadal development.

The *Sry* gene is the master regulator that drives testis differentiation [4]. An interesting question that remains unanswered is whether there is a similar master regulator gene that regulates brain sex differentiation. It has been argued that *Sry* originated from the brain determining gene Sry-type HMG box 3 (SOX3) [60]. Mutations in SOX3 cause an abnormal hypothalamic–pituitary–gonadal axis leading to male hypogonadism [61]. This suggests that the brain may harbor other important genes that regulate gonadal function and sex differentiation in a sex-specific manner.

The zebrafish is an ideal model system that holds promise to dissect the sex-specific differences in the brain as it offers several advantages over other models, including small size, short generation time, and easy gene manipulation. With recent advances in omics and gene editing technology, it has become possible to elucidate the signaling mechanisms in the zebrafish brain. Moreover, the zebrafish genome is well conserved with humans, and approximately 82% of the human disease genes are present in the zebrafish genome [62]. In some cases, zebrafish show higher conservation with humans than mice [62] and there is a high similarity between zebrafish and human brain organization [63]. Consequently, zebrafish has emerged as an outstanding model and its recent experimental use in neurological studies is significantly outnumbering those conducted in other model organisms [64].

There are many reviews on gonadal sex differentiation of zebrafish [20, 65, 66] and other teleosts [23, 67–69]; however, no systemic studies have summarized all available information on zebrafish brain sex differentiation. Hence, this study provides comprehensive information to further understand zebrafish brain sex differentiation and its associated challenges.

### Zebrafish brain sex differentiation

Similar to mammals, the molecular mechanism of brain sex differentiation in teleost fish is not fully understood. Among vertebrates, fish exhibit the most diverse sex-determination systems. Some fishes are reported to have XX/XY system, while others have ZW/ZZ system for sex differentiation [9, 70]. In zebrafish, the presence of sex chromosomes was debatable, as many studies have failed to find a sex chromosomes [71–74] and only two studies showed the presence of sex chromosomes in zebrafish, with females being heterogametic (ZW/ZZ system) [75, 76]. A study by Anderson et al. [77] revealed that this could be because sex chromosomal analysis has mostly been performed in domesticated strains. The authors working with the wild zebrafish collected from nature showed that there is polymorphism in chromosome 4 with females being heterogametic, suggesting a ZW/ZZ system [77]. The same polymorphism has not been found in the domesticated strains [77]. Consequently, the domesticated strain relies on multiple genes for gonadal differentiation and a polygenic sex determination system has been proposed [20, 78]. The administration of exogenous androgens and estrogens from the early developmental stages is known to skew the sex ratio in zebrafish [24, 25, 28]. However, sex reversal has also been reported in adult zebrafish, where an aromatase inhibitor treatment for 5 months in adult female zebrafish resulted in the retraction of the ovaries and formation of testis-like structures filled with spermatozoa-like cells [79]. In *foxl2*, *cyp17a1*, and *cyp19a1a* (gonadal aromatase) knockout zebrafish, defects in ovarian differentiation were observed, and an estradiol treatment could rescue the phenotype [38, 80, 81]. More specifically, *cyp19a1a* knockout in zebrafish resulted in all-male populations, and an estradiol treatment could rescue the phenotype by promoting ovarian development [80]. This suggests that zebrafish gonads are plastic even after sexual maturation, and steroid hormones or their genetic regulators are necessary to maintain the internal sex organs. Similar to gonads, the zebrafish brain also shows plasticity. For instance, exposure to 11-ketotestosterone (11-KT) in adult female fish can change the sexual behavior to that of male fish [82]. Using *cyp17a1* (which is involved in androgen synthesis) knockout zebrafish, we showed that a lack of androgen could lead to the development of an all-male population, and the mating behavior of the knockout males was also altered. A testosterone or 11-KT treatment on the *cyp17a1*-deficient fish at the adult stage restored the male-typical mating behaviors [83]. Germ cell numbers are also involved in brain sex differentiation, as the morpholino-mediated depletion of germ cells leads to an all-male population, but the brain transcript profile resembles that of the female brain transcript [29]. To date, steroid hormones have emerged as the main regulators of brain sex differentiation. However, the precise role of steroid hormones in organizing sex-specific neuronal networks remains unclear. Knockout studies of steroid hormone synthesizing genes have resulted in sex reversal; therefore, the direct effect of steroid hormones on the brain is unclear. The generation of brain-specific knockouts could help unravel the molecular mechanisms and key players involved in zebrafish brain sex differentiation.

The zebrafish is excellent for the study of different aspects of human physiology and has potential in unravelling the molecular mechanisms and etiology of human neurological diseases, including Alzheimer’s disease (AD), Parkinson’s disease (PD), etc. [62–64, 84]. Zebrafish can help predict the outcome as well as the mechanisms of action following gene and pharmacological intervention in the zebrafish brain. The molecular mechanism of zebrafish brain sex differentiation and sex-specific differences are essential to better understanding these neurological issues. There are some limitations with zebrafish as a neurodegenerative disease model has certain limitations; for instance, in contrast to mammals, adult zebrafish display extensive neurogenesis capability and can regenerate their brain following traumatic injury [85–88]. This could present an obstacle in understanding the progression of neurodegenerative diseases.

### Differences at the cellular level

Each brain cell type plays a specific role in maintaining brain homeostasis. For instance, microglia are known to regulate immune function, and sexual behavior, and they are also implicated in neurological diseases [89, 90]. Males and females are known to show sex-related differences in brain cell types: for instance, the rodent medial preoptic area that controls male sexual behavior has 2- to 3-fold more dendritic spines [91]. Microglia, the innate immune cells of the central nervous system, have been implicated to be involved in the shaping of neuronal networks. Female neonatal rat hippocampi show a higher number of microglia and phagocytic activity than the male hippocampi [92]. An analysis of the deposited RNA sequencing data showed that the microglia of mice have sex-specific gene expression [93]. Sex-specific differences at the cellular level have started to emerge in mammalian systems; however, similar information is lacking for the zebrafish model.

The adult zebrafish forebrain shows a sex-specific cell proliferation pattern; however, it is not known if these differences lead to differential neural organization [94]. It is also unknown at what stage this differential proliferation pattern is evident in zebrafish. Ampatzis et al. [94] used 5-bromo-2′-deoxyuridine (BrdU) and TUNEL assays to examine proliferative and apoptotic cells, and showed that the cell renewal properties in female zebrafish were higher in the medial zone of the telencephalic area, periventricular nucleus of the posterior tuberculum and the ventral part of the periventricular pretectal nucleus. In males, the dorsal zone of the periventricular hypothalamus showed higher activity [94]. The adult neurogenesis capability dictates neural plasticity; it has been suggested that fish species have higher plasticity than mammals. Apart from their high neurogenic potential, fish brains also have a high expression of aromatase (an enzyme that converts androgen to estrogens), which has been linked to neurogenesis [95]. In zebrafish, aromatase is localized in the radial glial cells of the forebrain, the pallial and subpallial regions, the preoptic area and the hypothalamus [96]. Radial glial cells are suggested to play a critical role in supporting the newly generated neurons by providing a scaffold for cell migration [97]. The radial cells are also shown to be the precursors of neuronal cells [98, 99]. Single-cell transcriptomics of male and female brains can further reveal critical sex-specific differences.

### Hormonal differences

Sex steroids are strong neuromodulators that can regulate behavior; in turn, altered behavior can change steroid levels. In teleosts, including zebrafish, 11-KT is a potent androgen [100–102]. In teleosts, sexual behavior is primarily regulated by gonadal steroid hormones [82], however, prostaglandins are also known to affect sexual behavior [103]. It was indicated that the sex steroids released by the female fish act as pheromones to attract males; however, it has also been shown that males can release pheromones as well to attract females for mating [104, 105]. In the protogynous grouper, the expression of genes involved in steroidogenesis can be detected in the brain during gonadal differentiation [106]. In rainbow trout, the expression of *cyp19a1b* (the gene that codes for brain aromatase) and *cyp11a1* (the gene that codes for the enzyme involved in converting cholesterol to steroids) is higher in male brains prior to gonad differentiation [107]. This suggests that steroid hormones in the brain can be produced prior to gonadal steroids in teleosts.

Hormonal analysis of the zebrafish brain showed that the E2 level is moderately high in the female brain, whereas, 11-KT is almost 2.5 times higher in males [29]. In another study, the testosterone levels were not significantly different between the male and female brains, but 11-KT was (average male level = 4725 pg/g and average female level = 91 pg/g) [83]. The brain can synthesize steroid hormones (also referred to as neurosteroids); however, how much of the measured steroid hormones are of the brain and gonadal origin is unclear. Different groups working on neurosteroids have shown that steroid hormones, including pregnenolone, dehydroepiandrosterone, and their sulfates are present in the mammalian brain [108–110]. Interestingly, these levels were maintained in the brain even after removing the adrenal and testis tissues. Using in vitro cell culture, Ruiz-Palmero et al. [111] demonstrated that neuronal cells can synthesize neurosteroids. Neurosteroid synthesis in the brain has also been observed in non-mammalian species, including birds and fish. Biochemical analysis revealed that zebrafish brain slices could convert pregnenolone to progesterone, suggesting a 3β-hydroxysteroid dehydrogenase isomerase activity [112].

Another sex hormone, progesterone, could also be important for brain functions, as in mammalian species; it acts as a pheromone [113] and promotes male and female sexual behavior [114–116]. The *progesterone receptor* (*pgr*) expression is sex-specific in the ventromedial nucleus of the hypothalamus (VMN) in rats [114]. The impact of progesterone on the sex-specific regulation of brain function has not yet been identified. However, progesterone exposure has been shown to negatively affect zebrafish reproduction and lower estradiol levels in females and testosterone and 11-KT levels in males [117]. Similar effects following exposure to synthetic progesterone, norethindrone, were also observed in other fish species, including fathead minnows and Japanese medaka [118]. In another study, a higher concentration of synthetic progesterone resulted in the masculinization of female fathead minnows [119]. This suggests that progesterone may also play an important role in the sex-specific regulation of zebrafish brain functions.

Using zebra finches (*Taeniopygia guttata*), Remage-Healey et al. [120] demonstrated that steroid levels in the forebrain fluctuate during social interactions. The fluctuation in the brain steroid levels was not related to the circulating levels, as the authors noted that when the male birds were given social stimuli, the circulating steroid level did not change but the estradiol level changed [120]. Recently, Nishiike et al. [121] reported that *estrogen receptor 2b* (*esr2b*) is the major determinant of sex-typical mating behavior and sexual preference in medaka, as the female medaka deficient in Esr2b are not receptive to males, but rather court females, despite retaining normal ovarian function with an unaltered sex steroid milieu. This emphasizes the role of the estradiol/Esr2b signaling pathway in determining sex-typical mating behaviors and sexual preference in teleosts. This is inconsistent with the *esr2b*-deficient female zebrafish, which showed normal mating and breeding behaviors [122]. The different observations in the *esr2b*-deficient medaka and zebrafish may be due to the divergent role of estrogen receptors in different species.

**Fig. 2:**
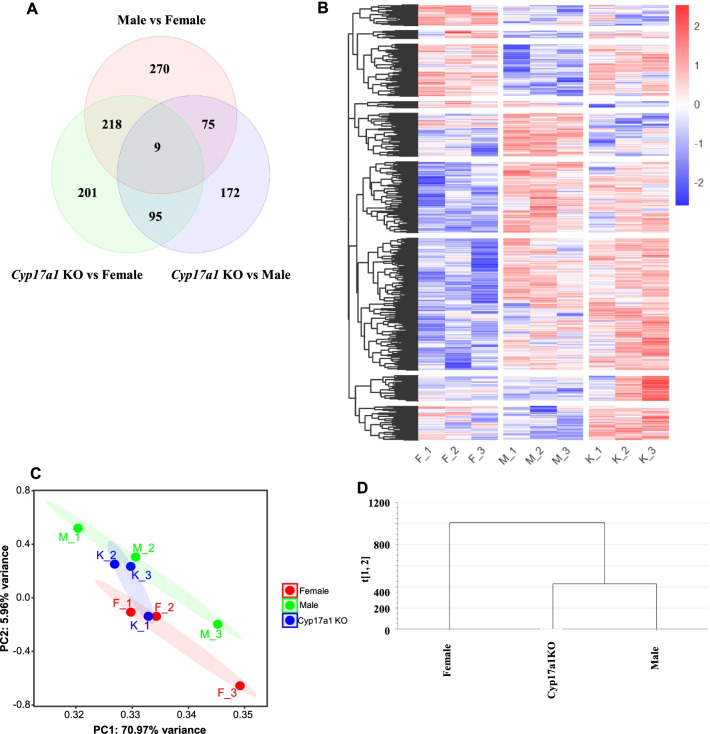
Comparison of female, male and *cyp17a1* KO brain transcriptomics. Zebrafish were maintained in a recirculating system in the Wuhan lab. Zebrafish at 90 dpf were anesthetized, and brain samples from wild-type females, wild-type males, and *cyp17a1* KO males were isolated. The sex was determined by examining the gonadal samples under the microscope. The brain samples were sent to NanJing Personal Gene Technology Co., Ltd., for transcriptomic analysis. Raw data were assessed for quality control using FastQC (v0.11.5). Adapter sequences were removed using Trim Galore (v0.4.3). The zebrafish reference genome (GRCz11/v104.11, Apr.2018) and the reference Index (the GTF file) were downloaded from Ensembl. First, hisat2-build was used to index the reference genome, and then HISAT2 (version 2.2.4) was used to map the reads to the reference genome. Finally, the gene counts were summarized with feature Counts (Subread software, v 2.03). The differential expression analysis was performed with the DESeq2 package (v1.30.1) using a fold change of 2 and a *p* value cutoff of 0.05. All the differentially expressed genes are presented in Additional file 1: Table S1. Hierarchical clustering of DEGs was performed in R (version 4.1.2) using the heatmap package. Venn diagram (**A**), heatmap (**B**), PCA (**C**), and dendrogram (**D**). Three independent biological replicates were used and for each biological replicate, four brain samples were pooled. F, female, M, male, K, *cyp17a1* KO

In our study of *cyp17a1* knockout zebrafish, we observed that androgen is important for gonadal sex differentiation as all the knockouts developed into males [38]. Although, the knockouts developed into males, the behavior and brain transcript analyses suggested that proper androgen levels are critical to fully masculinize the male brain [83]. We further performed transcriptomic analysis on the brain samples to determine whether the mutant male brain was similar to the wild-type female brain. We found 218 differentially expressed genes (DEGs) common between *cyp17a1* KO and male brains. Of these, *oat* (downregulated in the presence of testosterone) [123], *mos* and *syt13* (female enriched genes) [124] were significantly downregulated in both *cyp17a1* KO and male brains. Meanwhile, 75 DEGs were shared by female and *cyp17a1* KO brains (for the complete list of DEGs, refer Additional file 1: Table S1). Interestingly, we observed that genes, including *dio2* and *igf1* were significantly downregulated in female and *cyp17a1* KO brains compared to male brains. We further performed a principal component analysis to investigate whether there is a specific clustering among female, male and *cyp17a1* KO brain transcriptional profiles. We found that male and *cyp17a1* KO brains clustered together, whereas female brains showed a different clustering pattern (Fig. 2). Taken together, these findings indicate that the depletion of androgen impacts the brain transcriptomics; however, the overall transcript profile of the KO male fish is close to that of wild-type males. We anticipated that the profile would be very similar to that of females. We suggest that this may be due to the compensatory effect of another *cyp17a* gene (*cyp17a2*) in zebrafish.

Zebrafish also show sex-specific differences in the expression of steroidogenic genes during the day–night cycle. The expression of *cyp19a1b* (neural aromatase) and *cyp11b* peaks at night only in males [125]. This suggests that the hormonal levels have different peak periods, which should be considered during hormonal or gene expression analysis.

### Gene-level differences

The transcript analysis has revealed important sex-specific differences in the zebrafish brain. With the advancement of RNA sequencing and microarray techniques, it has become possible to screen for differences in almost the entire genome. Our lab has identified important genes that are expressed in a sex-specific manner in different regions of the male and female brains [82]. Among the 32 genes analyzed, 13 genes showed differential expression. The genes, including *cyp19a1b* (brain aromatase), *esr1*, *esr2b*, *mtf*, *ptgds*, *ptgs2b*, *sirt1* and *sod1* were highly expressed in females while *cfos*, *dio2*, *gabbr1a*, *gabbr1b* and *igf1* were highly expressed in males [82]. The sex-specific expression of *dio2* (*deiodinase type 2*) gene has been consistent in different studies [25, 29, 82, 83, 126]. The Dio2 enzyme is involved in the synthesis of thyroid hormone, it converts prohormone, thyroxine (T4) to the biologically potent hormone, triiodothyronine (T3). Thyroid hormone is involved in different aspects of brain development, as it regulates neurogenesis, neuronal migration, neuronal and glial differentiation, myelination and synaptogenesis [127, 128]. Thyroid hormone signaling has been found to be sex-specific in mouse brain [93, 129], but it is not known whether thyroid hormone is involved in induction of sex-specific signaling in the developing brain. The role of thyroid hormone in zebrafish is not entirely clear. In zebrafish, thyroid hormone disruption using goitrogen (methimazole) drives ovarian differentiation, whereas an exogenous treatment with T4 results in a male-biased population (sexing based on histological analysis) [130]. In a study by Houbrechts et al. [131] using whole-body *dio2* knockout zebrafish, it was observed that reduced thyroid hormone levels lowered the steroid hormones (T, E2, and 11-KT) in the gonads and delayed reproduction. Interestingly, our study showed that in the steroid hormone synthesizing gene *cytochrome P450, family 17, subfamily A1* (*cyp17a1*) knockout zebrafish, the *dio2* expression in the brain was downregulated threefold in both RNA sequencing and qPCR, while a rescue experiment with androgens upregulated *dio2* levels by twofold compared to the non-rescued fish [83]. This suggests that steroid and thyroid hormones are interlinked and that they are involved in the regulation of zebrafish brain sex differentiation. In contrast, *Dio2* knockout mice had normal T3 levels and unimpaired reproductive capacity, despite the disruption of thyroid stimulating hormone (TSH) feedback regulation, [132]. *Dio1* knockout mice also did not experience any effects on general health or reproductive capacity [133]. This suggests that in mice, Dio1 and Dio2 could compensate for each other’s loss.

**Fig. 3:**
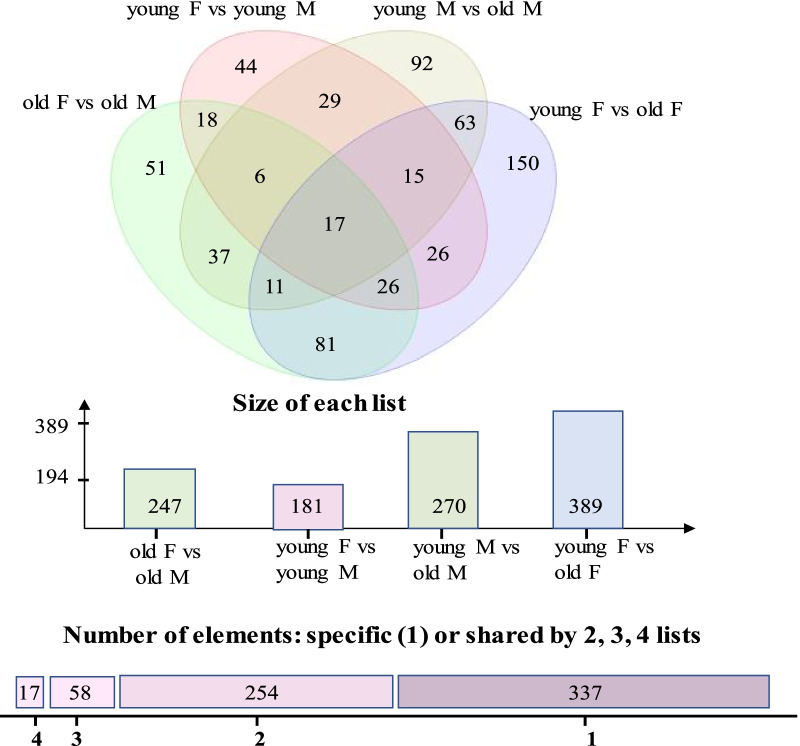
The zebrafish brain shows sex- and age-dependent differences. Zebrafish microarray data from NCBI were downloaded and analyzed using the Partek Genomic Suit software. The data suggest that both the young and old brain zebrafish brains show sex-specific differences. Within the same sex there was age-dependent gene expression. The raw data were obtained from a previously published study [130]

Depending on *cyp19a1b* expression, larvae can be segregated into high- and low-expression groups prior to gonadal differentiation [57–59], but these studies did not determine the sex of those two populations. Interestingly, some studies have shown high *cyp19a1b* expression in males [82, 126] and unbiased expression in the adult and juvenile zebrafish brains has also been reported [58, 134]. *cyp19a1b* expression was also not found to be sex-specific in our analysis using the deposited microarray data [135]. This suggests that *cyp19a1b* expression could be regulated by different factors and it could depend on the time of sample isolation, age, and strain. The microarray analysis of male and female brains of different age groups revealed that the sex-specific differences in the brain become more prominent as the fish mature [135]. In this study, the sex was determined by the visual observation of gonads, and fish without prominent eggs or testes were not used [135]. We analyzed the deposited microarray data [135], Gene Expression Omnibus (GSE53430) to further understand sex-specific differences. As indicated in the published study [135], we observed that male and female brains show distinct transcriptional profiles in both young (7.5- to 8.5-month-old fish) and adult (31- to 36-month-old fish) stages of life and start to be different from early on, but later in the adult stage, the differences are further noticeable (Fig. 3). We suggest that this could be due to the expression of different genes involved in steroid hormone biosynthesis in the male and female brains during the adult stage, as several genes including *sult2st2* (sulfotransferase activity in steroid hormone biosynthesis), *ugt1b5* (steroid hormone biosynthesis), *cga* (follicle hormone activity), *fkbp5* and *klf9* (glucocorticoid-responsive regulatory genes) [136–138] were differentially expressed in old female vs old male brains but not in young female vs young male brains. We further analyzed young female vs old female and young male vs old male transcriptomic data to understand whether steroid hormone biosynthesis-related genes differ during the adult stage in the same gender. We found that several steroid hormone biosynthesis-related genes such as *hsd17b3* (synthesis of 11-KT), *cyp39a1* (conversion of cholesterol to bile acid), *apof* (cholesterol transport and metabolic process), and *rspo1* (ovarian germ cell differentiation) [137, 139–141] were differentially expressed in old male brains compared to young male brains. The expression of *cyp27a1.4* (cholesterol metabolic process), *cyp2aa7* (steroid hydroxylase activity), *apoeb* (lipid transport), *cga* and *fkbp5* [136, 142, 143] were significantly different in old female brains compared to young female brains. Taken together, these data further confirm that male and female brains exhibit different transcriptomic patterns, and this difference becomes more distinctive during the adult stage.

The analysis of brain transcripts across four different strains revealed that only 61 genes showed sex-specific expression and out of these genes, 48 genes were highly expressed in males. A stronger effect was observed for genes involved in the steroid hormone pathway [126].

Apart from these molecular differences, a recent study indicated that epigenetic processes can also regulate masculinization and feminization processes in mammals [39]. DNA methylation patterns showed that new-born female rats had more methylated CpG sites than male rats. The DNA methyltransferase (Dnmt) activity was also lower in females, and the treatment of new-born females with estradiol resulted in decreased Dnmt activity. This suggests that the inhibition of masculinization through DNA methylation is important for brain feminization [39]. This indicates that epigenetic mechanisms could also be involved in prenatal brain for sex-specific gene expression.

In zebrafish, epigenetic mechanisms are known to alter gonadal sex differentiation. Ribas et al. [144] showed that exposure to the DNA methylation agent, 5-azacytidine can lead to the development of an all-female fish population. However, studies indicate that whether 5-azacytidine can drive the male or female pathway depends on the exposure concentration and time of exposure [145, 146]. Nevertheless, these studies show that the disruption of epigenetic mechanisms can alter the outcome of gonadal sex differentiation. The analysis of brain samples from the sex-changed zebrafish would have provided an important clue as to whether epigenetic changes also contributed to incomplete masculinization or feminization in sex-changed animals.

Male and female zebrafish brains showed sex-specific methylome patterns at 914 sites. In males 435 CpG sites were hypermethylated and 479 hypomethylated compared to females [147]. Of the 914 differentially methylated CpGs, 708 were found to be associated with protein-coding genes [147]. This suggests that epigenetic mechanisms in the zebrafish brain regulate important signaling processes that may also dictate brain sex differentiation. Epigenetic processes, including DNA methylation in the brain, play essential roles and altered functions are linked to neurodegenerative diseases [148]. Differential epigenetic mechanisms in males and females may also be responsible for the sex-specific neurological disease susceptibility. Hence, the further understanding of zebrafish brain epigenetic regulation could help dissect neurodegenerative diseases.

### Protein-level differences

It is indicated that around 50% of the human genome is expressed in the central nervous system, and the post-translational modifications and protein–protein interactions further increase the protein variants [149]. Alterations in protein structure, components and activity can lead to different neurodegenerative diseases, including amyotrophic sclerosis (ALS), Parkinson’s disease, and Alzheimer’s disease (AD) [150–153]. Understanding gene regulation at the protein level can help map signaling networks and neurodegenerative diseases. Many studies have identified sex-specific differences in zebrafish at the transcript level; however, there is limited information on these differences at the protein level. Recent advances in protein biochemistry have helped annotate proteins in zebrafish. Gabriel et al. [149] using two-dimensional electrophoresis and mass spectrophotometry (LC–ESI MS/MS) identified 95 different proteins in the adult zebrafish brain. The authors also showed that phosphorylation is a more common post-translational modification than glycosylation [149]. Sex-related differences at the protein level in the zebrafish brain were clearly shown in a recent study where the authors used hypoxia conditions to analyze changes in the brain proteome [154]. Among these proteins, H3k9 which is involved in epigenetic processes was significantly upregulated in male brain following hypoxia. Other proteins that were significantly altered were Eno1, Foxo1, Gp1, Hmox1, Nos2, Pkm, Ran, Vcp, Klf4, Nestin, Sox2, etc. Furthermore, pathway analysis showed clear sex-specific differences, especially in the disease and function categories [154].

### Metabolite-level differences

Metabolomics can reveal crucial information on the signaling mechanisms in male and female brains. Metabolomics involves the measurement of the end-products of cellular processes that can help elucidate biochemical processes, including neurodegenerative diseases [155]. The brain is mainly composed of lipids, which are associated with maintaining brain homeostasis and common pathological conditions in the brain [156]. As there is an alteration in lipid metabolism signaling in certain neurological diseases, including AD, the lipidomics of brain samples can provide essential clues on signaling processes [156]. The metabolic profile of the brain has been shown to be unique in different brain regions [155, 157], and the metabolomics profiling of the mouse brain has revealed that the metabolites are expressed in a region-specific manner [155]. For example, glycerophosphoserine was found to be high in the cerebellum and low in the frontal cortex, and another metabolite, *N*-acetylaspartylglutamic acid was found to be high in the brain stem and thalamus midbrain [155]. Hence, further analyses could shed light on the region-specific homeostasis during disease onset and progression.

Different factors, including age, genetics, lifestyle, and sex, can influence lipid metabolism in the brain. Testosterone and thyroid hormone are crucial hormones known to affect brain development, while they can also influence lipid metabolism [158, 159].

**Fig. 4:**
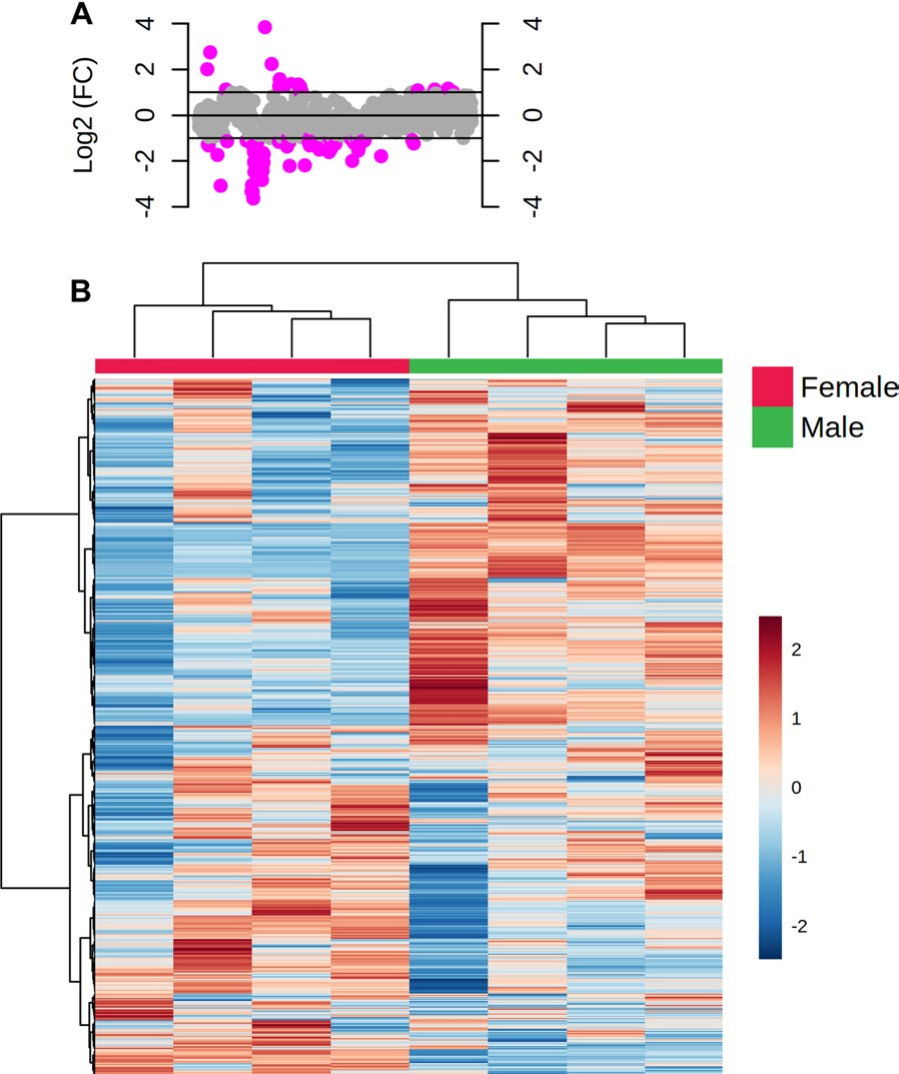
Metabolomics of male and female zebrafish brains. Control (WT; wild-type) male and female brains were analyzed for different lipid molecules. The fold change analysis indicated that there are 98 lipid molecules that are differentially produced (**A**). The heat map shows the overall difference (**B**) ANOVA, n = 4. The raw data were obtained from a previously published study [155]. In this study [155], the sex of the fish was determined by visual observation of the gonads

There is no clear understanding on sex-related differences at the metabolite level. To understand whether the zebrafish brain shows sex-specific differences, we analyzed the lipidomics data from a previous study [160]. The analyzed data suggests that metabolomic profile (lipidomics) in male and female zebrafish was different (Fig. 4). The raw data were obtained from a previous study in which control male and female differences were not reported [160]. Raw data was analyzed using the software MetaboAnalyst 5.0. The analysis showed that 98 lipid molecules in total were differentially produced in male and female brains (Fig. 4A). Out of 98 lipid molecules, 18 were highly produced in females, while 80 were highly produced in males (Fig. 4A, B). Different metabolites, including ceramide, cholesteryl palmitate, triacylglycerol, phosphatidylcholine, phosphatidylethanolamine, phosphatidylserine and sphingomyelin showed differential expression. Gene expression analysis (at the transcript level) has been the preferred method to show the sex-specific differences in the brain. However, understanding the differences in metabolites could further provide vital clues for deciphering differences in neuronal signaling networks.

## Conclusion

Zebrafish brains display sex-specific differences in terms of signaling and function. The differences are noticeable at the transcript, protein, hormonal, metabolite, and cellular levels. Although the primary determinant of brain sex differentiation has not been identified, the role of steroid hormones in the neuronal organization in a male and female typical manner is recognized. The zebrafish model can be an important tool to investigate how the sex-specific organization of the brain occurs and how different factors, including the genetic and environmental, influence the outcome. Deciphering the molecular mechanisms of brain sex differentiation could further help fill this knowledge gap and improve our understanding of neurological disease onset and progression. This can also help predict pharmacological interventions and bridge the gap between neurodegenerative drug discovery and clinical trials. This review discusses the essential differences that have been observed so far in male and female zebrafish brains. This information can be critical to target the candidate genes and signaling processes further to better understand the brain sex differentiation enigma.

## Supplementary Information

**Additional file 1.** List of differentially expressed genes for Fig. 2.

## Data Availability

Figure 2 transcriptomics raw data files are uploaded in NCBI database. Accession Number: SRP347573 and Bioproject: PRJNA783180.

## Abbreviations

11-KT: 11-Ketotestosterone
AD: Alzheimer’s disease
ALS: Amyotropic sclerosis
amhr2: Anti-Mullerian hormone receptor type 2
amhy: Y chromosome-linked anti-Müllerian hormone
apoeb: Apolipoprotein Eb
apof: Apolipoprotein F
AR: Androgen receptor
cga: Glycoprotein hormones, alpha polypeptide
cyp11a1: Cytochrome P450, family 11, subfamily A, polypeptide 1
Cyp17a1: Cytochrome P450, family 17, subfamily A1
cyp17a2: Cytochrome P450, family 17, subfamily A, polypeptide 2
cyp19a1a: Cytochrome P450, family 19, subfamily A, polypeptide 1a
cyp19a1b: Cytochrome P450, family 19, subfamily A, polypeptide 1b
cyp2aa7: Cytochrome P450, family 2, subfamily AA, polypeptide 7
cyp27a1.4: Cytochrome P450, family 27, subfamily A, polypeptide 1, gene 4
cyp39a1: Cytochrome P450, family 39, subfamily A, polypeptide 1
dio1: Deiodinase type 1
Dio2: Deiodinase type 2
dmrt1: Doublesex- and mab-3-related transcription factor 1
dmY: DM-domain gene on the Y chromosome
E2: Estradiol
Enp1: Essential nuclear protein 1
esr1: Estrogen receptor 1
esr2b: Estrogen receptor 2b
fkbp5: FKBP prolyl isomerase 5
foxl2: Forkhead box L2
foxo1: Forkhead box O1
gabbr1a: Gamma-aminobutyric acid (GABA) B receptor, 1a
gabbr1b: Gamma-aminobutyric acid (GABA) B receptor, 1b
gdf6Y: Growth differentiation factor 6 on the Y chromosome
Gp1: Glycoprotein 1
gsdfY: Gonadal soma derived growth factor on the Y chromosome
Hmox1: Heme oxygenase 1
hsd17b3: Hydroxysteroid (17-beta) dehydrogenase 3
igf1: Insulin-like growth factor 1
Klf4: Kruppel-like factor 4
KO: Knockout
mtf: Metal transcription factor
Nos2: Nitric oxide synthase 2
PD: Parkinson’s disease
Pkm: Pyruvate kinase, muscle
ptgds: Prostaglandin D2 synthase
ptgs2b: Prostaglandin-endoperoxide synthase 2b
Ran: Ras-related nuclear protein
rspo1: R-spondin 1
sdY: Sexually dimorphic on the Y chromosome
sirt1: Sirtuin 1
sod1: Superoxide dismutase 1
SOX: Sry-type HMG box
Sox2: SRY-box transcription factor 2
sox3Y: Sox3 on the Y chromosome
SRY: Sex determining region Y
T: Testosterone
T3: Triiodothyronine
T4: Thyroxine
TSH: Thyroid stimulating hormone
Vcn: Valosin containing protein
WT: Wild type
